# ORCA.IT: A New Web-Based Tool for Assessing Online Reading, Search and Comprehension Abilities in Students Reveals Effects of Gender, School Type and Reading Ability

**DOI:** 10.3389/fpsyg.2019.02433

**Published:** 2019-11-01

**Authors:** Martina Caccia, Marisa Giorgetti, Alessio Toraldo, Massimo Molteni, Daniela Sarti, Mirta Vernice, Maria Luisa Lorusso

**Affiliations:** ^1^Scientific Institute IRCCS Eugenio Medea, Bosisio Parini, Italy; ^2^Department of Brain and Behavioral Sciences, University of Pavia, Pavia, Italy; ^3^Milan Center for Neuroscience (NeuroMI), Milan, Italy; ^4^Carlo Besta Neurological Institute (IRCCS), Milan, Italy; ^5^Department of Psychology, University of Milano-Bicocca, Milan, Italy

**Keywords:** online reading comprehension, assessment, gender effects, education effects, reading difficulties

## Abstract

ORCA.IT, a new online test of online research and comprehension was developed for the Italian population. A group of 183 students attending various types of upper secondary schools in Northern Italy were tested with the new tool and underwent further cognitive and neuropsychological assessment. The different school types involved in the study are representative of the school population in the Italian system, but can also be easily compared with the educational systems of other countries. The new test turned out to have good psychometric properties after accurate item construction and final selection. In particular, Version 1 showed better characteristics than Version 2. Subsequently, comparison with one-way ANOVAs were performed to test whether differences exist between different school types, between groups with and without reading difficulties, and between males and females. Such differences are sometimes reported in the literature, but many remain controversial. Further, Pearson’s bivariate correlations were calculated to analyze associations between scores on the ORCA.IT and cognitive/neuropsychological variables. Finally, a stepwise regression analysis was performed on aggregated scores to identify the predictors of performance on each of the two versions. The test, especially in the most complete version (Version 1), appears to accurately and reliably capture students’ web searching abilities and online reading comprehension. The tool could highlight differences in online search and comprehension ability between students with and without reading difficulties, not penalizing overall performance but allowing very specific weaknesses to be pointed out. Further, it seems to be able to capture differences due to both educational pathways (different school types) and social attitudes (differences between males and females). Most interestingly, it shows to be clearly resting on specific cognitive and neuropsychological abilities, including language, memory, and attentional skills, which explain a large portion of the total variance. Offline text reading comprehension is a crucial predictor of online reading performance, while decoding ability is not. Prior knowledge also influences the results, as expected. The new tool turns out to be rather independent of previous Internet experience and to measure more cognitively grounded processes related to information gathering, processing, and communicating.

## Introduction

### ICT, Internet and Literacy

In the last years, there has been an increasing interest in how young people use the Internet and other new technologies in their everyday lives and how this use may enhance informal and formal learning opportunities (Becta, 2008). Indeed, previous research has shown that there is great variability in the ways they access and use Internet sources (Facer and Furlong, 2001; Livingstone and Helsper, 2007; Hargittai and Hinnant, 2008). In light of this, there is a need to better understand the complex factors determining this heterogeneity and what this may mean for the educational system (Selwyn, 2009). This constitutes one of the challenges for researchers, policy makers and teachers in order to use new technologies more effectively for formal education and develop more targeted initiatives that better support young people in their uses of the Internet and other information and communication technologies (ICTs) (Leu et al., 2011).

Nowadays, there is a tendency to promote ICTs adoption in many fields, including schools. However, many teachers make a poor and rare use of technology during their lessons and, usually, they do not fulfill the expected results (Korte and Hüsing, 2006; Shewbridge et al., 2006; Law et al., 2008; Leu et al., 2011; Davies and West, 2014; Fraillon et al., 2014). Leu et al. (2011) supported the idea that ICTs for educational purposes should take into account that the notion of literacy has now changed: it is now deictic; its nature and meaning continuously changes. Moreover, effective use of online information requires additional online reading comprehension practices. Public policy, assessment, and education should be able to prepare students for such challenges.

The arrival and spread of electronic resources and digital libraries have changed and extended the notion of literacy beyond its original application to the medium of writing. Nowadays, many researchers converged on the concept of “multiliteracies,” to define both the social diversity of contemporary forms of literacy, and new communications media and communicative competence (Cope and Kalantzis, 2000). “Digital literacy” perpetually changes because new technologies for information and communication constantly appear online and require new social practices of literacy.

The United Kingdom media regulator (Office of Communications [Ofcom], 2004) defined “Digital literacy” as the “ability to *access*, *understand*, and *create communications* in a variety of contexts.” *Access* refers to the skills needed to locate media content, using the available technologies (Buckingham, 2007). *Understand* takes into account the ability to decode or interpret media and it also involves knowledge of production processes and an ability to evaluate the specific media, for example, in terms of the accuracy or reliability of the web sources (Buckingham, 2007). Lastly, *create* consists of the ability to use the media to produce and communicate one’s own messages (Buckingham, 2007).

### Offline and Online Reading

As shown above, reading online implies high levels of critical skills; indeed Fabos (2004) stressed the importance of promoting more critical analysis of online content. Actually, online reading comprehension is a process which requires to analyze many different sources of online information, using several recursive reading practices (Coiro, 2003; Henry, 2006), following what is now known as the LESC model (Leu et al., 2013): (i) reading to locate information (L); (ii) reading to evaluate information (E); (iii) reading to synthesize information (S); and (iv) reading and writing to communicate information (C). Specifically, online reading requires both new online and traditional offline reading comprehension skills (Leu et al., 2011).

Reading and searching online information usually implies that a question has been formulated. Taboada and Guthrie (2006) identified differences, within traditional texts, between reading that was, or was not, initiated by a question. Moreover, online reading is a multi-componential process and requires, for example, the generation of effective keyword search strategies (Bilal, 2000; Kuiper et al., 2008), inference as to which link may be most useful within a set of search engine results (Henry, 2006), and efficient evaluation of relevant information within websites (McDonald and Stevenson, 1996; Rouet, 2006). Successful online reading requires also to tell reliable information from fake news (Sanchez et al., 2006; Graesser et al., 2007). However, such practices present challenges that are quite different from those regarding traditional print and media sources, because the content of online information is more assorted and commercially biased than that of print sources (Fabos, 2008; Leu et al., 2011). Online Reading Comprehension also requires the ability to synthesize information from multiple sources (Jenkins, 2006) and communicate and discuss it via the Internet (Britt and Gabrys, 2001; Kiili et al., 2012).

Online and offline reading skills are organized in complex ways (Leu et al., 2015) and they share some similarities (Coiro, 2011; Hahnel et al., 2016). During reading comprehension, prior knowledge plays an important role in building a logical representation of the text (Kintsch, 1998). An additional factor that may play a role, especially when one reads about controversial issues (as it often happens during online searching), is a reader’s prior beliefs on an issue. This may influence text interpretation (Nickerson, 1998) or website evaluation (van Strien et al., 2016).

Kiili et al. (2018), in their study on the students’ abilities to critically evaluate online sources, found that offline reading skills were necessary but not sufficient for a successful evaluation of online resources; they hypothesized that offline reading does not involve the same amount of critical evaluation skills that is required in online reading. Moreover, to the authors, poorer offline readers seem to show more difficulties in online evaluation, suggesting that offline reading skills are necessary for a successful online critical evaluation.

However, other studies found a lack of a strong relationship between offline reading practices and online reading evaluation. It appears that online and offline reading comprehension are different processes (Coiro and Dobler, 2007; Afflerbach and Cho, 2010) actually, a study by Bråten et al. (2009) showed that online critical evaluation skills appear to be separate and independent of offline reading skills.

### Factors Underlying Individual Differences in Online Reading

Among the context-dependent and experiential factors that may influence online reading abilities, special attention has been devoted to familiarity with the topics. As mentioned above, prior knowledge of the topic plays an important role in the comprehension of the different types of texts (Cromley et al., 2010; Tarchi, 2010) and hypertexts (Amadieu et al., 2009). However, Coiro (2011) found that even though prior topic knowledge played an important role in online research and comprehension performance of students with low online reading skills, it did not influence the performance of students with high online reading skills. Moreover, Kanniainen et al. (2019) found that the relationship between prior knowledge and Online Reading Comprehension was not significant.

Indeed, students may feel inadequate in assessing sites when they are unfamiliar with their topics. They largely fail to apply reliable criteria; rather, they emphasize (e.g.) speedy access to information and appealing visual design (Buckingham, 2010). In their research, Eynon and Malmberg (2011) investigated five uses of the Internet: communicating, information seeking, entertainment, participating, and creating, among young people. They found four different Internet user profiles: the peripherals, the normatives, the all-rounders, and the active participants. The first group was the least frequent users of the Internet, tending to do less of all Internet activities than the other groups and showing a lack of skills to use it. The normatives showed average uses of three types of Internet activity – communicating, entertaining, and information seeking – and were engaged less in more proactive uses of the Internet such as creating and sharing actively contents. The all-rounders used the Internet for all five types of Internet use more frequently than the average. Active participants are those who use the Internet most frequently, for all five activities, and tend to engage in online participatory behaviors (like contributing to a blog or wiki page) more frequently.

Familiarity with specific topics, as well as with specific approaches to information collection and analysis may further depend on the students’ educational paths. In the Italian system there are three main types of secondary schools: Lyceum, Technical Schools, and Vocational Schools. These types of schools differ with respect to specific curricula, as well as to a more theory-oriented or more practice-oriented approach, and these differences may entail different habits with respect to information search (how and where to look for information) and processing (how to handle it). To our knowledge, no previous study focused specifically on the influence by school type on students’ Internet usage. In their study on Internet inequality, Zhao et al. (2010) found that students with available digital devices at home tend to have the strongest perception of Internet skill. Availability of social support from school has a greater effects than that from home on Internet self efficacy (ISE), which describes learners’ confidence in their general ability to operate Internet functions or applications in Internet-based learning conditions (Tsai et al., 2011). A higher ISE has been shown to be associated with better information search strategies, better learning skills, and better learning performance, while more controversial results have emerged on the relationship between ISE and actual Internet usage or navigational paths (Tsai et al., 2011). Zhao et al. (2010) further found that school Internet accessibility seems not to be significantly related to ISE. According to the authors, one reason might be that in most high schools of China, the Internet-related resources, such as computers with access to the Internet, are inadequate. As to the Italian context, in spite of the strong pressure from the Ministry of Education to update and upgrade ICT resources in the schools (e.g., a wider use of MIW – Multimedia Interactive Whiteboards, and Classrooms 2.0, as fostered by the Piano Nazionale Scuola Digitale – National Plan for Digital Education in response to OECD requirements, Miur, 2015), equipments are very often old or poorly maintained and teachers are often not familiar with the new technologies (Gremigni, 2019); moreover, the students’ abilities in searching information in the Internet and evaluating its reliability are still lower than the OECD average (Miur, 2015). Overall, it would thus be difficult to predict whether different school types differ with respect to the opportunities they offer to their students to reach a good mastery of online search and comprehension skills. Indeed, more theory-oriented schools probably require frequent comparison of different sources (but not necessarily through the Internet); at the same time, more practically oriented schools may use digital tools also for technical purposes (e.g., graphics software), which could improve students’ familiarity and confidence with ICT tools.

Following the previous literature, other factors may concur to reading comprehension, specifically, Snowling (2013) suggested that students with low verbal and non-verbal reasoning skills are more likely to have comprehension difficulties. Non-verbal reasoning has been shown to have direct and indirect effects on reading comprehension (Swart et al., 2017) and, in line with this, Kanniainen et al. (2019) found that non-verbal reasoning contributed independently to the variance of Online Reading Comprehension performance.

Reading abilities are clearly good candidates to play a central role in online reading, as suggested by the relationships between offline and online reading skills. However, very little is known about the behaviors of people with dyslexia in web usage. This is probably due to the general focus on the consequences that decoding difficulties have on school performance in younger readers, and on the belief that decoding ability only plays a major role in beginning reading acquisition, and is subsequently replaced by comprehension skills. Indeed, it is now evident that the distinction between decoding and comprehension is less clear-cut than previously thought (e.g., Gough and Tunmer, 1986), the two kinds of difficulty interacting with each other and being frequently present in the same individual, possibly at different time points during development (Bishop and Snowling, 2004; García and Cain, 2014). This may clearly apply to online reading too. Very little is known, though, about the behaviors of people with dyslexia in web usage. McCarthy and Swierenga (2010), in their review, argued that dyslexic-friendly practices may help overcome difficulties faced by all Internet users. In 2004, the United Kingdom’s Digital Rights Commission conducted a task-oriented examination of 100 websites and a group of people with dyslexia took part in the study. Each user completed two tasks on ten different websites. Dyslexic users experienced a 17% failure rate (which was lower than the rate experienced by – for example – blind and partially sighted participants). The main issues experienced by dyslexic users were: confusing page layout, unclear navigation, poor color selection, difficulties in decoding graphics and complicated language.

Kurniawan and Conroy (2007) tested reading comprehension speed and accuracy during Internet gathering information of dyslexic and non-dyslexic students and they found that participants with dyslexia made increasingly frequent mistakes as reading material became more complex but allowing users to select their ideal color scheme increased reading speed for both groups. A general idea emerging from the literature is that dyslexia is highly variable; actually there is no “typical” dyslexic Internet user. Pollak (2001) interviewed college students with dyslexia and he found that they underline the potential strengths of multimodal documents with respect to unimodal ones.

It is well established that many students with dyslexia also experience language or visual-spatial difficulties (Giovagnoli et al., 2016; Snowling and Melby-Lervåg, 2016), and that their visual-verbal integration skills may also be poor (Hahn et al., 2014). This means that complex text comprehension may be problematic, even when the coding difficulties have been largely overcome or compensated, and this may be true not only for written text, but also for complex oral explanations that accompany online videos, or graph interpretation. Such difficulties may be more evident in high school, when texts are more often supported by non-textual materials, and may be especially relevant for the multi-media and multi-modal information that is found in the Internet.

A final interesting issue in literacy research is gender-related differences. Several studies showed an advantage for girls in reading fluency and reading comprehension (Logan and Johnston, 2009, 2010; Torppa et al., 2018) and similar patterns have also been observed in other studies on Online Reading Comprehension (Forzani, 2016; Salmerón et al., 2018; Kanniainen et al., 2019). McKenna et al. (2012) reported that middle school girls have more positive reading attitudes toward reading print texts for recreational as well as academic purposes, while their attitudes toward reading digital texts are better for academic purposes, but not for recreational purposes. A similar finding is reported by Lupo et al. (2017) with high school students. Meelissen and Drent (2008) propose that this may be due to female students showing less positive attitudes toward using computers as compared to male students. By contrast, no significant differences emerged between females and males in a Korean middle school sample (Jang and Ryoo, 2018) in academic-related Internet activities. The authors propose that this may result from the strong achievement-driven characteristics of Asian secondary schools.

### Effects of Web Usage on Cognitive Functions

The ready availability of information on the Internet may decrease the need to store and recall data. Sparrow et al. (2011) suggested that people may be becoming better at remembering where information is located than at recalling it; this has been defined as the “Google effect.” It has been suggested that individuals born after 1993 (the so-called “Google generation”) may show weaker working memory and be less confident about their answers as compared with older individuals, even if they retrieve information and make responses more rapidly (Nicholas et al., 2011). Moreover, Dong and Potenza (2015) showed in their study that even if Internet-based searching may have facilitated the information-acquisition process, this process may have been performed more rapidly and be more likely associated with difficulties in recollection. In addition, people appeared less confident in recalling information learned through Internet searching.

Online searching seems to have changed our attentional abilities. In their study, Ophir et al. (2009) explored the impact of the sustained media multi-tasking on cognitive skills. They found that frequent and extensive (called “heavy”) media multi-tasking performed worse in task-switching tests than the normal multi-media users. It was suggested that the compromised ability in heavy media multi-tasking people was due to their increased proneness to distraction from irrelevant environmental inputs. However, literature on multi-media and Internet usage have produced conflicting findings (Firth et al., 2019). Nevertheless, on the whole, the literature seems to agree on the fact that those who engage in frequent and extensive media multi-tasking in their daily lives perform worse in various cognitive tasks than those who do not, particularly for sustained attention (Uncapher and Wagner, 2018). Moreover, a longitudinal study of media multi-tasking in young people has found that frequent multi-tasking behaviors predict the development of attentional deficits specifically in early adolescents but not in older ones (Baumgartner et al., 2017).

### The Present Study

The present study is outlined using an online research and comprehension framework (Leu et al., 2011), which focused on the four crucial component skills mentioned above (LESC, i.e., Locate, Evaluate, Synthesize, and Communicate) and on different types of media (texts, images, videos, and graphs). Specifically, students’ online reading abilities were measured with “ORCA.IT,” an Italian adaptation of the online research and comprehension assessment (ORCA) originally developed in the ORCA Project (Coiro and Kennedy, 2011; Leu et al., 2014). Several ORCA tools had been developed in this Project (Leu et al., 2014). ORCA.IT was inspired by ORCA-Multiple Choice. This was a performance-based assessment within a more restricted and limited simulation of the Internet. In this format, students were guided through a research task by a student avatar that contacted them through a social network, and another student avatar that contacted them through instant messaging, all within the ORCA space. Topics were: Energy drinks and heart health, Videogames and effects on eyes, Use of decorative lenses and effects on eyes, Safe volume levels for Ipods. Students used fully functional tools (a social network, text (chat), email, wikis, a search engine, and a notepad) to conduct their research. Italian ORCA was designed to emulate a natural online research process even if it did not use a fully functioning simulation of the Internet (such as social networks, wikis, avatars, and the possibility to write texts). Students were asked to use Internet tools (a search engine, email etc.) to conduct their research on a specific given topic within a simulation of the Internet. All questions required to choose among pre-constructed responses (either verbal responses or images). The topics included in the Italian version (Electromagnetic waves and health and Music and brain) were different from those addressed in the original version and had been chosen so as to be edge-cutting, likely interesting to the students and not part of any standard school science program and/or curriculum. The skill areas that were evaluated (LESC) did not appear in a strictly linear sequence in any of the two topics. Also, differently from the original version and due to specific hypotheses about the impact of different communication modalities on performance (especially for students with special needs and different reading abilities), the Italian version distinguished between items that belong to four different typologies: verbal texts, graphs, images, and videos. Separate scores could be calculated for each of these areas, and a profile of each student could be visualized at the end of the test, showing the areas of relative strength and weakness, along with graphs depicting the LESC/typology profile for the single students as compared to her/his classmates (class averages).

ORCA.IT, in a similar way to the original ORCA project, assesses and considers the student’s pre-existing knowledge of each topic. In the Italian version only, however, questions on prior knowledge included also a whole section devoted to previous experience with ICT tools and habitual use of multimedia technology. Throughout the assessment of both prior knowledge and the two topics, the multiple choice format allowed all the scores to be calculated automatically through implemented algorithms. Thus, differently from the original test, the Italian test does not require any additional judgment or scoring by the teachers or examiners.

Finally, the Italian device includes an original feature that is meant to support reading by students with reading difficulties: Text To Speech with a natural Italian female voice, that can be activated by the student for any part of the texts to be read (instructions, questions, answers, embedded texts, and parts of the graphs).

Summing up, our research questions were:

1. What are the general psychometric characteristics of the new test? In other words, is the test able to capture the students’ abilities in a valid and reliable manner?
2. Are online reading abilities related to offline reading skills?
3. What are the predictors and components of online reading abilities (digital experience, prior knowledge, offline reading comprehension, STM, WM, and executive skills)?
4. How do students’ online reading abilities differ by school type, gender, and reading (dis)ability status?

Based on previous studies and specific hypotheses (concerning the nature of the teaching approaches, which is more theory-focused in lyceum studies, more practice-based in vocational schools, and with intermediate characteristics in technical schools), lyceum students were expected to score generally higher than technical school students, who in turn were expected to score higher than vocational school students. However, non-textual information (especially interpretation of graphs and still images: indeed, videos require good oral language comprehension abilities) was expected to show smaller differences between school types as compared to text-based information; students with reading difficulties were expected to score lower than students without reading difficulties – although, in this case too, differences could be expected to emerge for textual information (and, possibly, video) only; males were expected to score higher than females, as found in previous research; this advantage was expected to concern non-textual more than textual information. Finally, statistical analyses were expected to reveal contributions from offline text comprehension abilities, prior knowledge, decoding abilities, attentional, and executive functions as expressed by visual search/selection tasks and questionnaires on attention and concentration abilities.

## Materials and Methods

### Participants

Participants were 183 students (53% boys and 47% girls) all attending the upper secondary school in four different provinces of Lombardy (northern Italy); schools were selected from diverse regions of Lombardy so as to cover a wide range of SES profiles. Students were recruited from three different school types/levels: Scientific Lyceum (35%), Technical School (43%), and Vocational School (22%). These curricula differed for gender distribution (χ^2^ = 14.18; *p* < 0.001), with girls preferring the Technical Schools (51%), and boys preferring Scientific Lyceum (48%). Age ranged from 14 to 17 years (*M* = 15.84, *SD* = 0.72). Inclusion criteria were: adequate socio-educational conditions and absence of neurosensory deficits or cognitive impairment (IQ ≥ 85 as assessed by either the Cattell Culture-fair test, Cattell and Cattell, 1981 or the Raven Standard Progressive Matrices, SPM, Raven, 2003). Fourteen students had a formal diagnosis of Specific Learning Disorders formulated by experienced clinicians, based on standard diagnostic criteria (ICD-10). Seven students were classified as having reading difficulties based on their actual performance on the Reading tests described below. Criteria for this classification were 2 SDs below the mean on at least one parameter (speed or accuracy in either word on non-word reading), or 1.6 SDs below the mean in at least two parameters. The sample also included 12 bilingual students, who had complete mastery of the Italian language. Bilingual students with insufficient mastery of Italian were excluded.

After receiving the school-manager’s approval to carry out the research, the caregivers and the students were informed on the aim and procedure of the study. Parents provided a written consent for their children’s participation in the study and students gave informed written consent to the study, according to the General Data Protection Regulation (GDPR 2016/79, 25/05/2018). Students completed the questionnaires and the tests in two group sessions and their decoding ability was assessed in one individual session. The present study was approved by the Scientific and Ethics Committee of the Department of Psychology of the Catholic University of Milan, in accordance with the Helsinki Declaration.

### Standardized Tests

All participants were administered with the following standardized tests (individually or collectively), which are the commonly used tests for assessment of Specific Reading Disorders in Italy.

#### Reading

Single Word/Non-word Reading: “Batteria per la Valutazione della Dislessia e della Disortografia Evolutiva, DDE-2” (Battery for the assessment of Developmental Reading and Spelling Disorders), by Sartori et al. (2007). This test assesses speed (in syllables per second) and accuracy in reading word lists (four lists of 24 words each) and non-word lists (three lists of 16 non-words each) and was standardized on high school students (Arina et al., 2013). Concurrent validity for the DDE-2 was assessed through correlations between word and non-word lists from this and another widely used test, on a sample of primary and middle school students: correlations between word lists is 0.96, between non-word lists it is 0.79. Reliability was assessed through test-retest procedures (0.77 for reading speed and 0.56 for errors) and through correlations among subtests (average correlation = 0.79).

#### Written Spelling

Spelling accuracy was assessed with a write-to-dictation task. A short text was read aloud by one examiner, in a clear and neutral voice, without emphasizing the source of the spelling difficulty and without explanations about words or expressions (Cornoldi et al., 2017). Accuracy is expressed by the number of errors made by the student. Substantial errors like omissions, inversions and substitutions are assigned one point (maximum 1-point per word). Standard scores are then calculated based on age norms. Correlation between word and non-word written spelling is 0.685.

#### Comprehension

This was evaluated using Italian texts appropriate for the student’s age (Cornoldi et al., 2010). The task requires silent reading followed by answering ten multiple-choice questions. The ability to extract the exact meaning from the text and to examine the information contained in a sentence is assessed. Internal consistency of the test for the second class is adequate (Cronbach’s alpha = 0.84).

#### Memory

Verbal short-term/working memory was measured by means of the digit span task from the Wechsler batteries (WISC–IV Wechsler, 2003). The experimenter reads aloud lists of single digits, and the participant is asked to recall them immediately after the end of the examiner’s reading, either in the same order (forward) or in reverse order (backward). One point is assigned for each sequence of digits correctly recalled; the number of digits in each sequence increases (by one digit) if the participant has correctly recalled at least one of two sequences of a given length. Administration is discontinued when both items from a given pair are failed. The sum of points for each subtest represents the total score for that subtest. Then, age-corrected weighted scores are calculated according to age norms. Internal consistency of the scales is satisfactory (for age 15, reported reliability is 0.87; for age 16, reliability is 0.85).

#### Attention

(a) The Brown Adolescent attentional disorder (ADD) Scale (Brown, 1996) is a self-assessment tool whose overall score is an indicator of the likelihood that the individual has some ADD; ADD probability is stratified in three categories: (i) possible but unlikely (overall score <40), (ii) likely but uncertain (40–54); (iii) very likely (>54). The Brown ADD subscales target subclinical impairments of executive functioning that impact academic, social, emotional and behavioral functioning. The adolescent version features five clusters frequently associated with ADD:

• Organizing, Prioritizing, and Activating to Work;
• Focusing, Sustaining, and Shifting Attention to Tasks;
• Regulating Alertness, Sustaining Effort, and Processing Speed;
• Managing Frustration and Modulating Emotions;
• Utilizing Working Memory and Accessing Recall.

As to reliability, the item-total correlation in the non-clinical Italian adolescent sample (average of the five clusters) equals 0.86.

(b) The d2-R Test is a neuropsychological measure of selective and sustained attention and visual scanning speed (D2-R; Brickenkamp et al., 2010). It is a paper-and-pencil test on which the participant is asked to scan some lines of letters and cross out all occurrences of the “d” letter while ignoring all other letters. D2-R provides a variety of measures, including Processing Speed, Accuracy of visual scanning, Coordination of speed and accuracy. Reliability estimates for the target age group are very high (Correct answers = 0.89; attention Performance = 0.90; Error percent = 0.91).

#### Language

(a) The Italian version of the Peabody Pictures Vocabulary Scale (Dunn and Dunn, 1997) was used to assess receptive lexicon. Additionally, (b) a newly developed online sentence comprehension test developed by one of the authors (Vernice et al., 2019) was proposed to assess receptive syntax. The test is based on 20 multiple choice trials. Each trial involves a target sentence of varying syntactic complexity that has to be read silently. The four sentences from which the response has to be chosen, include: a paraphrase of the target sentence (correct choice), a sentence contradicting the meaning of the target sentence, a sentence compatible with the target sentence, but not equivalent to it, and a distractor with different content. The score is the total number of equivalent sentences detected. The sentence comprehension test was presented using Google forms. No reliability data are available at present.

### Materials

An online platform has been developed to test students’ ability to gather, comprehend, evaluate, synthesize, and report on information, to conduct research in order to answer questions or solve problems through new media forms and Internet.

The online reading comprehension test was delivered through a specially designed web-app. The web-app was developed using Ruby on Rails + Vue.js, one of the JavaScript frameworks for web applications, used for the realization of graphical interfaces. The interface is designed for use on tablets and desktops.

**FIGURE 1:**
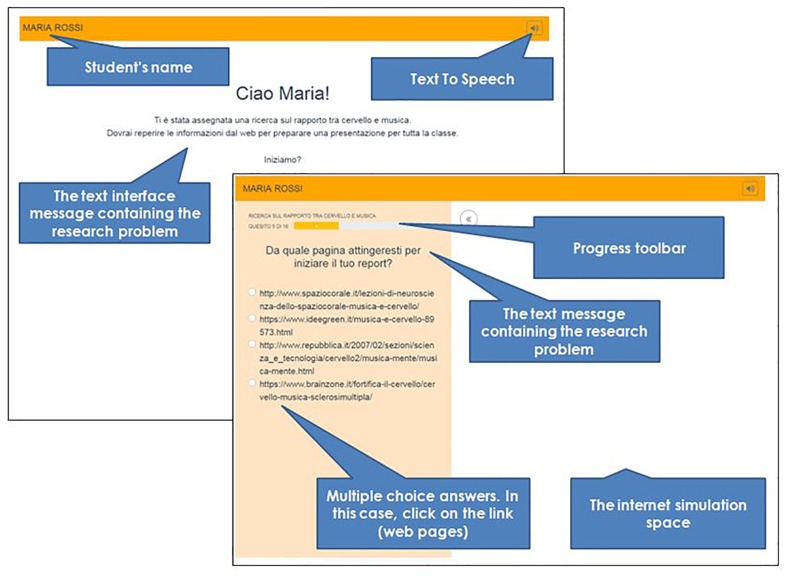
Structure of the ORCA.IT online test.

The graphics is friendly and simple (see Figure 1). The computer screen is divided into two parts: the left side of the screen contains the multiple-choice questions, while the right part is the Internet simulation space.

The device includes *Text To Speech* with a natural Italian female voice. It was selected for the quality of the Italian voice^1^. The button off/on was integrated in the form, and by clicking on it, it is possible to listen to any text element on the page.

The device has been designed according to the theoretical framework described in the introduction which identifies four crucial component skills: (1) locating information, (2) evaluating information critically, (3) synthesizing information, and (4) communicating information. These component skills have been assessed through questions addressing two different topics: (a) Music, Brain, and Neurosciences; (b) Electromagnetic Pollution and Health. Such topics were considered to be interesting for adolescents and not straightly linked to school content.

The students are required to plan the correct steps and select the best elements within a fictional context where they are requested to build a report on a given topic exploring online resources (news web pages with different credibility and reliability) and to distinguish relevant online resources from irrelevant ones or from potential fake news. Component skills (LESC) were tested with 19 multiple-choice (four options) questions for topic (a) and topic (b).

**FIGURE 2:**
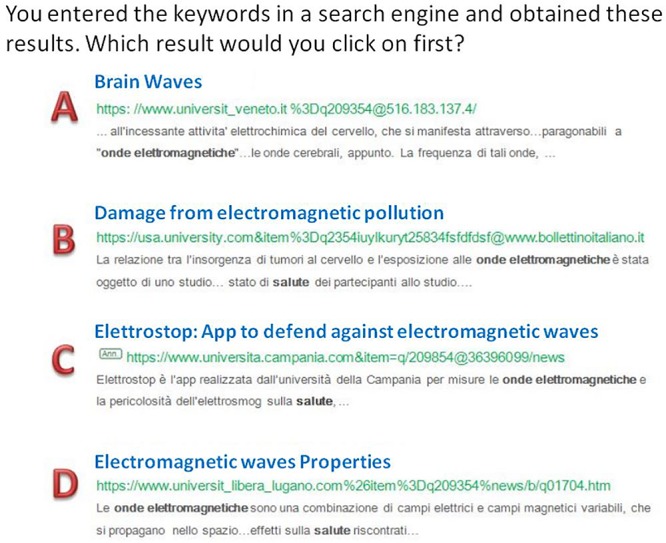
Example of a question with the four multiple-choice answers. The question was preceded by an introductory item: “You have been requested to write a report on the relationship between electromagnetic waves and health. You will have to find information on the web and prepare a presentation for your classmates” and the student had chosen the keywords for the search. Answer A was assigned 0 points (unrelated topics) as well as answer C (advertisement); answer B was assigned 2 points, and answer D 1 point (correct but less complete topics).

The answer options included one correct option, two incorrect options, and a partially correct option. Two points were given for each correct choice, one point for a partially correct choice and zero points were given for incorrect options. See Figure 2 for an example. A partially correct option is an answer that captures some, but not all, of the relevant aspects or elements; alternatively it may be a relatively good option, but clearly less appropriate than the full-score option (accordance between different judges had been assessed through blinded assignment of scores and subsequent comparison, with an inter-rater agreement of about 0.87). As an exception, questions where only one correct answer was possible did not envisage partially correct (1-point) options, and thus included three incorrect (0-point) and one correct option (2-points).

Questions were grouped around the four LESC components described for online reading comprehension, i.e., (1) Locating information, (2) Evaluating information critically, (3) Synthesizing information, and (4) Communicating information. This allowed subscale total scores to be automatically calculated for each component.

Further, all questions were classified according to the type of materials used to convey information, distinguishing among Texts, Images, Videos, and Graphs. Also for these four categories it was thus possible to automatically calculate total subscale scores. Even if watching a video or analyzing a graph certainly activate multiple processes that need to be integrated following complex pathways, we decided to classify also multi-media (or multi-modal) and non-verbal items within the LESC model. As a result, all questions concurred in producing the scores for both the LESC and the “type of media” subdivision.

Prior to the proper ORCA.IT test, three subtests for prior knowledge (one concerning Internet use and two concerning the topics of the ORCA.IT test) were delivered as parts of the total assessment session (and part of the software). The three subtests are described below.

### Digital Skills

Familiarity and competence/skills in different forms of digital literacy was subsequently assessed. The test covered three main dimensions of digital literacy: preference and frequency of use of digital instruments, operational skills (needed to operate computers and network hardware and software), and self-evaluation skills. The items from the three sections are inspired by DigComp 2.0 (The Digital Competence Framework for Citizens, Vuorikari et al., 2016) that identifies the key components of digital competence. The students responded using a 5-point Likert scale (from 0 = “not at all/never” to 5 = “very much/very often”) for all items.

### Prior Knowledge

Then, students were required to answer questions assessing Prior Knowledge, with respect to the two topics: (a) Music, Brain, and Neurosciences; (b) Electromagnetic Pollution and Health. The first question investigated the level of familiarity with the research topic. The following questions concerned specific information on the subject. For each research topic, there were five further multiple-choice questions. A total score was automatically calculated for topic (a) and for topic (b).

### ORCA.IT

Two equivalent versions of the ORCA.IT test were designed, intended as two parallel versions of the test for repeated testing. The general structure of the tests was the same for both versions, but the specific questions varied. More specifically, the same questions that on one version were formulated using topic (a), were reformulated for the other version based on topic (b). The number of questions for each component of the model (Locate, Evaluate, Synthesize, and Communicate online information) and text-image-graph-video structure was also maintained equal for the two versions. Digital skills and Prior Knowledge questions are exactly the same in the two versions. For each version there were 38 items: 19 items on topic (a) and 19 items on topic (b).

The fourth LESC component skill – Communicating information – assessing ability to address a specific audience (teacher or schoolmates), in each version of ORCA.IT, was assessed by introducing the request to either prepare a report and write an email, or to prepare a slide presentation to show the results. These tasks were always accomplished by choosing among a series of four different formats; each of the questions tapped different aspects of communication skills and strategies. Both the “email communication” mode and the “report in Power Point” mode is available in Version 1 and Version 2, albeit on different topics.

The sequential composition of the two versions is described below:

**Version 1** – Digital skills; Prior Knowledge (Music and Brain); questions based on Topic (a) (first part); Prior Knowledge (Electromagnetic Pollution); questions based on Topic (b) (second part).
**Version 2** – Digital skills; Prior Knowledge (Music and Brain); questions based on Topic (a) (second part); Prior Knowledge (Electromagnetic Pollution); questions based on Topic (b) (first part).

### Procedure

The test was administered in the months of April and May 2019. On two separate days, students were administered all tests described above (reading, writing, text comprehension, memory, and cognitive tests). At least 2 months elapsed between the Standardized Tests and online reading comprehension assessment (ORCA.IT). The administration of ORCA.IT was done in the computer room and each student operated on computer (laptop or desktop computers). Completion of the online test required about 45 min. Cognitive and neuropsychological testing required about 1 h for collective testing and 20 min for individual testing. All testing was performed by licensed psychologists.

In a selection of schools, students were required to undergo testing with both Version 1 and Version 2. A total of 44 students belonged to this group. The aim was to assess the equivalence of the two versions. One half of the students took first Version 1 and then Version 2; for the other half, the opposite order was followed. Only the scores obtained in the version that was taken first were included in the data to be analyzed, so as to avoid learning or tiredness effects. The scores obtained on the version taken second were used for comparison purposes only.

## Results

### Pilot Studies

The scores collected from the pilot study (*N* = 30) were analyzed item-by item, in order to identify ceiling and floor effects. As a result, about 20% of the 38 initial items were either reformulated so as to facilitate item comprehension, or eliminated when more than 90% of the students gave correct responses. Moreover, eight new items were added to the list, so as to have a larger range of items to choose from in a later selection.

A second pilot study was run (*N* = 10) in order to confirm that the new items had a better distribution. Following this step, six more items were replaced by new ones with clearer formulation and/or more challenging content.

### Final Tests and Subject Selection

The final test included 29 items (plus five items for prior knowledge assessment) for both Version 1 and Version 2. These were administered, respectively, to 79 and 89 subjects. Since each of the 29 items had a forced-choice four-alternative structure, in which the correct response was granted two points, the overall score ranged 0–58. Subjects’ mean scores (±*SD*) were 35.1 ± 7.2 for Version 1 and 32.2 ± 6.8 for Version 2. At this stage, we tackled the issue of chance level – that is, subjects should be excluded who, due to low motivation, did not perform the test properly and selected responses at random. A simple Monte Carlo simulation study (*N* = 4,000) confirmed that chance level was 19/58 points (which can be mathematically derived from the scores granted by each item), and found a standard deviation of 4.562. Since the shape of this simulated score distribution was almost perfectly Gaussian, we learnt that 95% of scores obtained by selecting responses completely at random would be below 26.5/58. Hence, setting a cut-off of score at 26.5 should exclude the vast majority of subjects who did not follow the instructions, in any of the items. There were indeed some subjects who did so: the cut-off led to the exclusion of 9 out of 79 subjects (11.4%) who were tested with Version 1, and 19 out of 89 subjects (21.3%) who were tested with Version 2. Any further analysis was carried out without those subjects.

### Psychometric Analysis, Item Selection and New Scores

Before performing inferential analyses, the general psychometric characteristics of the test were analyzed. Test coherence as measured by Cronbach’s Alpha turned out to be rather low for both versions: 0.514 for Version 1 and 0.361 for Version 2. Thus, we selected items in order to achieve a Cronbach’s Alpha of at least 0.6. First, we excluded all items showing a negative correlation with total scale score. Then we performed a stepwise procedure: at each step, Alpha was re-computed after having left out one of the items; the item associated with the highest increase in Alpha was excluded, and the procedure repeated on a new step. If on a same step two or more items led to the same increase in Alpha, the choice was made relying on the quality of the distribution of the items. This led Version 1 to be reduced to 24 items (Cronbach’s Alpha = 0.605). Excluded items were A1_3, A1_11, A1_15, B2_9 and B2_13. Inter-item correlations ranged from −0.25 to 0.471; mean correlation was 0.063. Mean value (±*SD*) for the new scale was 30.52 ± 6.27. Inter-class correlation was 0.060, *F*(70,1610) against 0 = 2.532, *p* < 0.001. For Version 2, it was not possible to reach Alpha = 0.6 even after reducing items to as few as 15, so it was decided to keep the 17-item version. In it, no item had a negative correlation with the total scale score and Alpha (equaling 0.548) had reached a plateau – further exclusion of items produces negligible increase of Alpha. The total score for Version 2 had mean (±*SD*) = 19.97 ± 5.1.

Aggregate scores were computed for each version based on the new selection of items, Vers1TOT and Vers2TOT. Aggregate scores for the various subscales were computed for Version 1 only (the reduced Version 2 had too few items in some of the subscales). These sub-scale specific scores were computed both as raw sums, and as percent of maximum possible score. Thus, for the LESC components, we obtained percent scores Lperc1, Eperc1, Sperc1, and Cperc1. Finally, a score was computed for each of the modalities with which the information was conveyed, again both as raw sums and as percent of the maximum possible score (Textperc1, Graphperc1, Imageperc1, and Videoperc1).

All scores of Version 1, and the total aggregate score of Version 2 underwent the statistical analyses we had planned for the whole sample, which are reported below.

### Tests of Specific Predictions/Questions

First of all, a general analysis of the distributional properties of the various variables and scales was performed, revealing sufficiently close-to-normal distributions for all variables.

Subsequently, planned analyses were run, yielding the following results.

#### Comparison Between Different School Types

**FIGURE 3:**
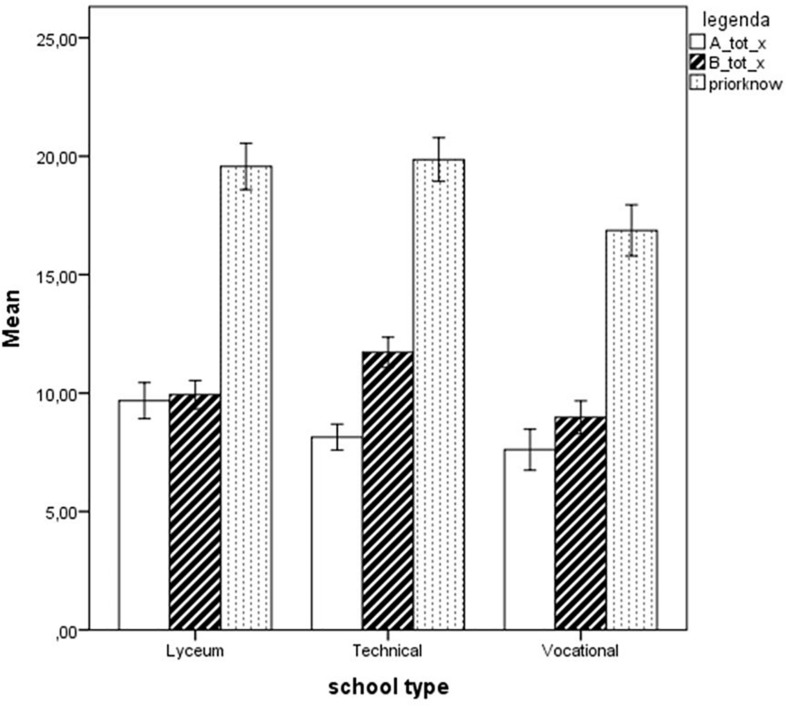
Performance of students from the three different school types on the two topics A (music and brain) and B (electromagnetic waves and health), and total prior knowledge (the sum of the two). Error bars illustrate 95% Confidence Intervals for the Mean.

Firstly, the three school types were compared with one-way ANOVAs with respect to the two pre-tests of ORCA.IT, i.e., the sections testing their digital and web-surfing abilities and their prior knowledge on the two topics addressed in the test. Students from the three school types obtained very similar scores on the questionnaires on digital competence and web-surfing habits and skills. However, their prior knowledge on the two topics differed, *F*(2,175) = 8.397, *p* < 0.001. Tukey’s post-test highlighted that the difference was due to students of Vocational schools scoring lower than both students of Technical schools (*p* < 0.001) and Lyceum (*p* = 0.002). A further analysis with repeated-measures ANOVA comparing the two types of topics (intra-subject factor) and the three schools (inter-subject factor) revealed that a significant main effect of School type [*F*(1,173) = 8.397, *p* < 0.001, partial η^2^ = 0.088] and of Topic [*F*(1,173) = 35.376, *p* < 0.001, partial η^2^ = 0.170] but also a significant School type by Topic interaction [*F*(1,173) = 16.014, *p* < 0.001, partial η^2^ = 0.156]. This was due to students of Lyceum yielding better scores compared to students of Vocational schools on the questionnaire on Topic a (brain and music) and students of Technical schools yielding better scores on topic b questionnaires (electromagnetic waves and health). These effects are shown in Figure 3.

**FIGURE 4:**
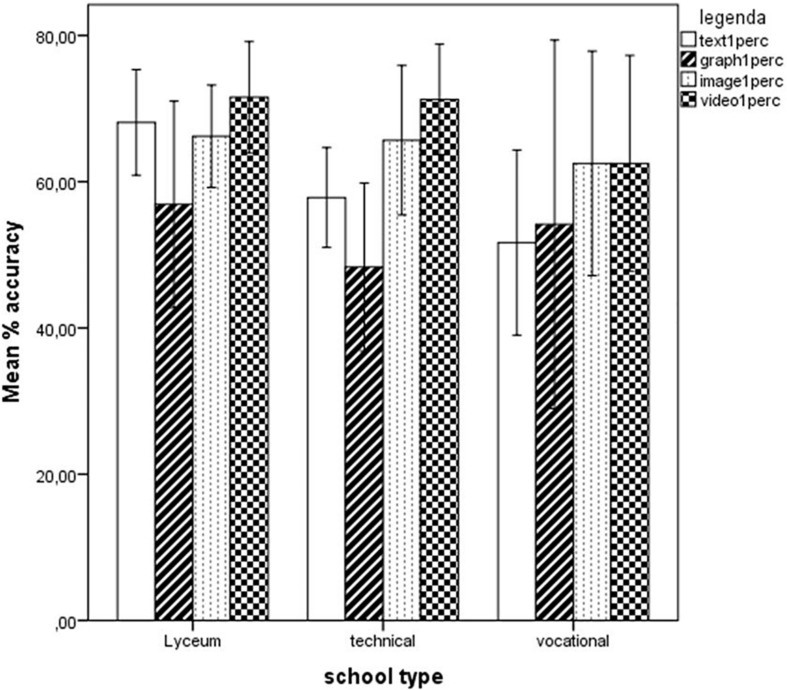
Performance of students from the three different school types on the four subscales Text, Graph, Image, and Video, expressed as percent scores. Error bars illustrate 95% Confidence Intervals for the Mean.

One-way ANOVAs were carried out on Version 1’s total score and its various subscales L1, E1, S1, C1 as well as Text1, Graph1, Image1, and Video1, with School type (three levels: Lyceum, Technical, and Vocational school) as a factor. No significant differences emerged between the different school types, with the exception of the aggregate scale Text1perc, part of Version 1, *F*(2,68) = 3.96, *p* = 0.024, partial η^2^ = 0.104. Tukey’s *post hoc* test revealed that the difference was due to students of Lyceum (*M* = 68.10, *SD* = 18.96) scoring higher than students of Vocational Schools (*M* = 51.67, *SD* = 19.92), *p* = 0.035 (see Figure 4).

#### Comparison Between Groups With and Without Reading Difficulties (RD, no-RD)

**FIGURE 5:**
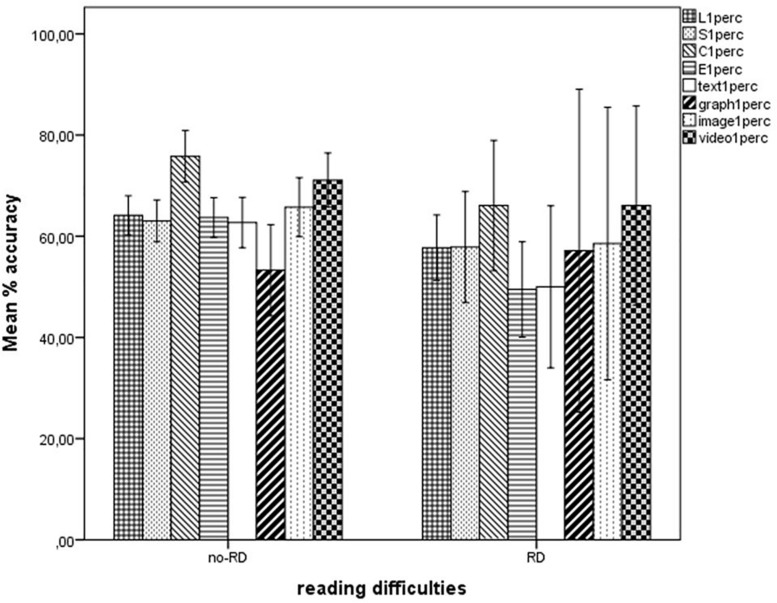
Results obtained by students with and without reading difficulties on the four subscales Locate, Evaluate, Synthesize, and Communicate and on the four types of materials Text, Graph, Image, and Video, expressed as percent scores. RD, reading difficulties. Error bars illustrate 95% Confidence Intervals for the Mean.

Some significant differences emerged when comparing the two groups (non-parametric tests were preferred, because in this case there were only seven subjects with reading difficulties). These were found with the aggregated score of Version 1 (Mann–Whitney’s *U* = 10.5, *Z* = −2.29, *p* = 0.022, mean rank 36.35 versus 18.36 for students without and with reading difficulties, respectively), and particularly in the Evaluate percent scale (Mann–Whitney’s *U* = 91, *Z* = −2.48, *p* = 0.013, mean rank 36.51 versus 17) and in the Text information percent scale (Mann–Whitney’s *U* = 119.5, *Z* = −1.905, *one-tailed p* = 0.029, mean rank 36.04 versus 21.07). These results are illustrated in Figure 5.

#### Comparison Between Males and Females

**FIGURE 6:**
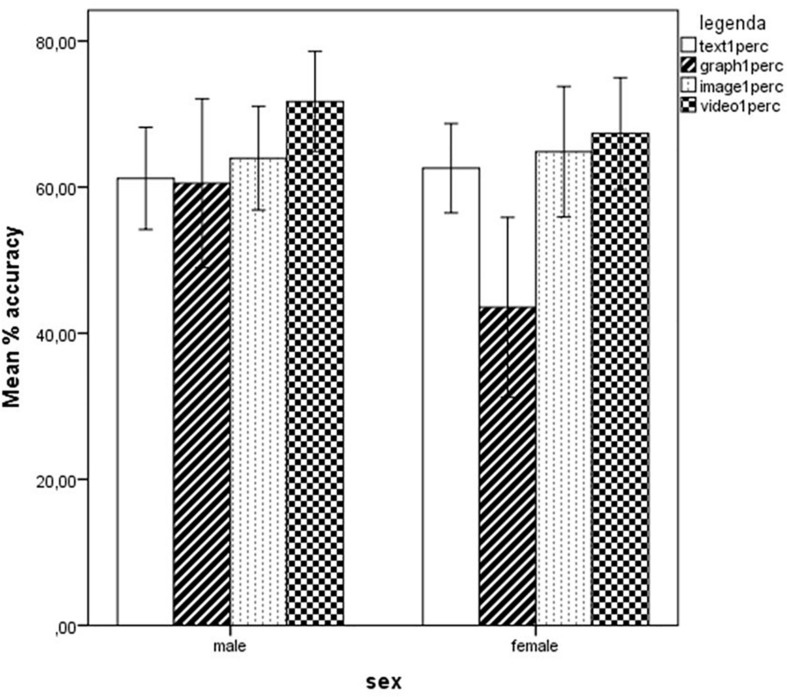
Results obtained by males and females on the four subscales Text, Graph, Image, and Video, expressed as percent scores. Error bars illustrate 95% Confidence Intervals for the Mean.

No significant differences emerged from either version between the two sexes, with the exception of the Graph scale, where males (*M* = 60.53, *SD* = 35.18) scored higher than females (*M* = 43.55, *SD* = 33.52), *F*(1,68) = 4.147, *p* = 0.046, partial η^2^ = 0.058 (see Figure 6).

#### Correlations Between the Aggregate Scores and Performances on Cognitive-Neuropsychological Tests

First of all, the scores obtained on all cognitive and neuropsychological tests were compared in the two groups of students who had been administered Version 1 and Version 2. No significant differences emerged in any of the variables (all *p*_s_ > 0.05).

Then, Pearson’s bivariate correlation indices were calculated to analyze associations between the variables. *Z*-scores were considered whenever available for the standardized tests, so as to partial out the effects of (small) age differences among the students. Significant correlations are reported in bold in Table 1.

**TABLE 1:** Pearson correlations between ORCA.IT variables and prior knowledge (digital competence and familiarity with topics), IQ, language skills (vocabulary and syntactic abilities), attentional variables (expressed by both objective and self-reported measures concerning organization capacity, attentional shifting and focusing, sustained attention, concentration, self-management, and emotional control), reading (words and non-words, speed, and accuracy), and memory measures (forward and backward digit span).

	**Version 1 total**	**L1**	**S1**	**C1**	**E1**	**Text 1**	**Graph 1**	**Image 1**	**Video 1**
PRIOR KNOWL	**0.367^∗∗^**	**0.355^∗∗^**	**0.424^∗∗^**	**0.088**	**0.405^∗∗^**	**0.315^∗∗^**	0.131	0.114	0.222
	*0.002*	*0.002*	*0.000*	*0.466*	*0.000*	*0.007*	*0.275*	*0.344*	*0.063*
	(71)	(71)	(71)	(71)	(71)	(71)	(71)	(71)	(71)
IQ	**0.285^∗^**	0.227	0.200	0.212	**0.244^∗^**	**0.309^∗∗^**	0.005	0.137	0.186
	*0.018*	*0.060*	*0.100*	*0.080*	*0.044*	*0.010*	*0.964*	*0.260*	*0.127*
	(69)	(69)	(69)	(69)	(69)	(69)	(69)	(69)	(69)
FW D_SPAN	**0.272^∗^**	0.138	0.109	0.236	0.155	**0.259^∗^**	**0.255^∗^**	−0.115	0.160
	*0.025*	*0.262*	*0.375*	*0.053*	*0.208*	*0.033*	*0.036*	*0.352*	*0.192*
	(68)	(68)	(68)	(68)	(68)	(68)	(68)	(68)	(68)
BW D_SPAN	0.235	0.107	0.182	**0.281^∗^**	0.116	**0.286^∗^**	−0.067	0.014	0.049
	*0.054*	*0.387*	*0.137*	*0.020*	*0.348*	*0.018*	*0.587*	*0.911*	*0.692*
	(68)	(68)	(68)	(68)	(68)	(68)	(68)	(68)	(68)
OFFL TEXT COMPR	**0.414^∗∗^**	**0.337^∗∗^**	**0.347^∗∗^**	0.090	**0.430^∗∗^**	0.228	−0.054	**0.391^∗∗^**	**0.251^∗^**
	<*0.001*	*0.005*	*0.003*	*0.463*	<*0.001*	*0.060*	*0.660*	*0.001*	*0.037*
	(69)	(69)	(69)	(69)	(69)	(69)	(69)	(69)	(69)
PEABODY	**0.379^∗^**	**0.343^∗^**	**0.382^∗∗^**	**0.295^∗^**	**0.416^∗∗^**	**0.315^∗^**	−0.076	**0.328^∗^**	**0.350^∗^**
	*0.010*	*0.021*	*0.010*	*0.049*	*0.005*	*0.035*	*0.622*	*0.028*	*0.018*
	(45)	(45)	(45)	(45)	(45)	(45)	(45)	(45)	(45)
SYNTAX	0.263	0.281	0.208	0.031	0.318^∗^	0.232	−**0.326^∗^**	**0.385^∗∗^**	0.237
	*0.077*	*0.058*	*0.165*	*0.838*	*0.031*	*0.121*	*0.027*	*0.008*	*0.112*
	(46)	(46)	(46)	(46)	(46)	(46)	(46)	(46)	(46)
NW_READ_ACC	**0.243^∗^**	0.041	0.037	0.135	**0.268^∗^**	**0.308^∗^**	−0.174	0.125	0.020
	*0.044*	*0.739*	*0.761*	*0.269*	*0.026*	*0.010*	*0.154*	*0.304*	*0.870*
	(69)	(69)	(69)	(69)	(69)	(69)	(69)	(69)	(69)
ATTENTION_CORR_RESP	**0.252**	0.165	0.170	0.156	**0.256**	**0.251**	−0.131	0.216	0.149
	*0.081*	*0.257*	*0.242*	*0.284*	*0.075*	*0.082*	*0.369*	*0.137*	*0.307*
	(49)	(49)	(49)	(49)	(49)	(49)	(49)	(49)	(49)
ATTENTION_OMISS	−0.053	0.155	0.034	0.022	−0.141	−**0.260**	−0.010	0.055	0.191
	*0.719*	*0.287*	*0.819*	*0.878*	*0.332*	*0.071*	*0.946*	*0.710*	*0.190*
	(49)	(49)	(49)	(49)	(49)	(49)	(49)	(49)	(49)
ATTENTION_CONC	0.184	0.012	0.115	0.073	0.250	**0.311^∗^**	−0.096	0.103	0.011
	*0.206*	*0.933*	*0.433*	*0.620*	*0.084*	*0.030*	*0.511*	*0.479*	*0.938*
	(49)	(49)	(49)	(49)	(49)	(49)	(49)	(49)	(49)
SR_ATT_CONC	−**0.302^∗^**	−0.178	−**0.296^∗^**	−0.164	−**0.283^∗^**	−**0.310^∗^**	−0.050	−0.171	−0.063
	*0.035*	*0.221*	*0.039*	*0.261*	*0.049*	*0.030*	*0.731*	*0.240*	*0.669*
	(49)	(49)	(49)	(49)	(49)	(49)	(49)	(49)	(49)

*KNOWL, knowledge; FW D_SPAN, forward digit span; BW D_SPAN, backward digit span; OFFL TEXT COMPR, offline text comprehension; NW_READ_ACC, non-word reading accuracy; ATTENTION_CORR_RESP, attention test – correct responses; ATTENTION_OMISS, attention test – omissions; ATTENTION_CONC, attention test – concentration; SR_ATT_CONC, self-reported attention test – concentration. Significant correlations are reported in bold, *p*-values in *italics*, N in parentheses.
*

#### Regression Analysis

A stepwise regression analysis was performed on total scores Vers1TOT and Vers2TOT to identify the predictors of performance on each of the two versions, based on the results of correlation analysis and *a priori* expectations. Criteria for entering the regression equation related to probability of F: *p* < 0.05 for entering, *p* ≥ 0.1 for removal.

Predictors were entered in three subsequent steps: IQ was entered first, followed by all subscales related to web use (frequency, competence, technical abilities, and surfing habits) and prior knowledge; then all other potential predictors were entered, more precisely language skills (vocabulary and syntactic abilities), attentional variables (expressed by both objective and self-reported measures concerning organization capacity, attentional shifting and focusing, sustained attention, concentration, self-management and emotional control), reading (words and non-words, speed, and accuracy), and memory measures (forward and backward digit span). Due to missing scores for the neuropsychological tests, 43 participants out of 71 were included for Version 1 and 51 out of 77 were included for Version 2.

For Version 1 (reduced to 24 item), seven variables were included in the equation in subsequent steps: IQ (explaining 13.6% of total variance), prior knowledge (20.9%), Offline reading comprehension (9.6%), self-reported attentional skills related to Working Memory (5.9%), Forward Digit span (7.2%), attentional skills related to Concentration (6.4%), self-reported attentional skills related to concentration (4.4%); overall, these predictors explained 68% of variance, *F*(7,40) = 9.999, *p* < 0.001. No multicollinearity issues were present (VIF ranged between 1.131 and 1.924).

For Version 2 (reduced to 17 items), four variables entered the regression equation: IQ (explaining 9.4% of total variance), attention/concentration scores (14.9%), non-word reading accuracy (7.5%) syntax (6%). Overall, 38% of total variance was explained, *F*(4,50) = 6.993, *p* < 0.001. No multicollinearity issues were present (VIF ranged between 1.019 and 1.493).

The coefficients for the two regression equations are reported in Tables 2, 3.

**TABLE 2:** Coefficients of the regression equation for Version 1 total score (24 items).

	**Non-standardized coefficients**		**Standardized coefficients**	***t***	***p*-value**
	**B**	**Standard Error**	**Beta**		
(Constant)	–21.335	10.883		–1.96	0.058
IQ	0.095	0.085	0.133	1.128	0.268
PRIOR KNOWL	0.524	0.183	0.299	2.857	0.007
OFFL TEXT COMPR	1.534	0.507	0.35	3.023	0.005
SR_ATT_WM	1.027	0.253	0.531	4.064	< 0.001
FWD_SPAN	1.473	0.416	0.378	3.544	0.001
ATTENTION_CONC	0.047	0.016	0.352	2.951	0.006
SR_ATT_CONC	–0.395	0.186	–0.29	–2.12	0.042

*KNOWL, knowledge; OFFL TEXT COMPR, offline text comprehension; SR_ATT_WM, self-reported attention test – working memory; FW D_SPAN, forward digit span; ATTENTION_CONC, attention test – concentration; SR_ATT_CONC, self-reported attention test – concentration.*

**TABLE 3:** Coefficients of the regression equation for Version 2 total score (17 items).

	**Non-standardized coefficients**		**Standardized coefficients**	***t***	***p*-value**
	**B**	**Error Standard Deviation**	**Beta**		
(Constant)	–13.987	8.891		–1.573	0.123
IQ	0.113	0.094	0.169	1.195	0.238
ATTENTION_CONC	0.046	0.013	0.411	3.501	0.001
NW_READ_ACC	–1.305	0.499	–0.309	–2.616	0.012
SYNTAX	1.095	0.518	0.300	2.114	0.040

*ATTENTION_CONC, attention test, concentration; NW_READ_ACC, non-word reading accuracy.*

## Discussion

The study described a new online test of online research and comprehension developed for the Italian population, that was named ORCA.IT.

Several questions, mostly regarding a number of *a priori* predictions, were addressed, which are reported and discussed in the following, under separate subheadings.

### What Are the General Psychometric Characteristics of the New Test?

The new test turned out to have good psychometric properties after accurate item construction and final selection. In particular, Version 1 showed better characteristics, with Skewness and Kurtosis for total score distribution being 0.073 and 0.723, respectively. The total scale was subdivided into subscales reflecting the structure that had inspired item construction, i.e., the so-called LESC structure (Locate, Evaluate, Synthesize, and Communicate online information). Also these subscales have good psychometric properties with close-to-normal distributions. As to Version 2, it was found to have poorer internal consistency and it was reduced to a 17-item scale in order to improve it, thus reaching sufficient reliability in terms of inter-item correlations, item-to-total scale correlations and Cronbach’s Alpha. For this reason, no subscales were computed and the scale cannot be considered as an equivalent, parallel version of Version 1, but rather as a different, shorter version with different characteristics.

### Are There Any Differences Between the Different Types of School?

The different school types involved in the study are representative of the school population in the Italian system, but can also be easily compared with the educational systems of other countries, with more theory-focused schools (Lyceum, here represented by a scientific and by a sport + science curriculum), technical schools (here represented by graphics and chemistry curricula), and more practice-oriented, vocational schools (here represented by commercial, education, and mechanics curricula). The three types of schools did not reveal clear differences with respect to students’ general performance on the ORCA.IT test. This is encouraging since it suggests that the different educational pathways have provided similarly effective (though certainly different in content) opportunities to develop online search and reading abilities. Furthermore, students from the three types of schools with different educational and professional orientation were found not to differ in digital skills (on digital competence and web-surfing habits and skills). This preliminary data is comforting if we consider the choice of the school as an expression of a socio-economic condition. In fact, research has pointed to correlations between the users’ socio-economic and cultural background and their use of ICTs (Gui and Argentin, 2011; Buffardi and Taddeo, 2017). Indeed, the failure to acquire digital skills can reproduce or even increase existing social inequalities (Hargittai, 2008; Bracciale and Mingo, 2015; van Deursen and Helsper, 2015). It is probable that the greater familiarity and greater exposure to written information (text) or the opportunities provided by the socio-cultural background can account for much of this difference. Differences between school types in Italy have also been found with regard to reading speed. In other words, the ability to read and comprehend offline has sometimes been found to significantly differ between school types, with poorer performance emerging in vocational institutes compared to lyceums (Stella and Tintoni, 2007). What is suggested by the present results, by contrast, is that online reading and web-based activities may have the potential to minimize such differences and reduce the gap in career opportunities and professional and personal satisfaction.

Nonetheless, differences across school types emerged in the qualitative profiles. Specifically, the comprehension and processing of textual information was found to be more difficult for students of the Vocational Schools. This may reflect a preference by students with higher verbal abilities to choose more theory-oriented schools, and vice versa, for students with lower verbal but higher practical skills to choose Vocational schools. Indeed, this is also supported by the much higher percentage of students with a diagnosis of Specific Reading Disorders in Vocational (and Technical) schools as compared to Lyceum where they are rare. It is also interesting to note that students from Technical schools were found to have even higher levels of prior knowledge on technical – oriented topics, whereas students from Lyceum were more familiar with more scientific topics, reflecting the relevance of the specific topic rather than of general abilities and general education on performance on the ORCA.IT test. In other terms, the two topics, which are both represented in both versions of the test, seem to have been effective in balancing and reducing the effects of possible specific prior educational differences. Clearly, as shown also by regression analysis, prior knowledge does have an influence on performance: this is in line with most previous studies (though not all those examining performance on ORCA instruments) and with our hypotheses, and a complete elimination of such effects would probably be both non-realistic and inappropriate.

### Are There Any Differences Between Males and Females?

Generally, males and females showed similar performances. Males were found to score better than females on a specific subscale only, i.e., the Graph subscale of Version 1. This might be related more to the usually reported higher ability of males on visual-spatial tasks than to specific digital skills or web-based search and comprehension skills. Similarly, in a study on Singapore secondary school students (Wu, 2004), male students performed better in graph reading, female students in graph construction, whereas no gender differences were found in graph interpretation and evaluation. While a general advantage for males in online reading performance cannot be documented due to the very little difference found in our sample (with marginal statistical significance), it can rather certainly be excluded that a general advantage for females (described for instance by Kanniainen et al., 2019 or Forzani, 2016) can be found in the Italian school system when accessing and using digital information. This, following Jang and Ryoo’s (2018) suggestions, might reflect a less positive attitude toward digital media in Italian girls, even for academic purposes (different, in this case, from both North-European and Asian female students). Moreover, a similar advantage for boys in digital reading had been described also by Rasmusson and Åberg-Bengtsson (2015), who put this in relation with the habit for males (more than for females) to spend many hours in playing with digital games, a difference that likely characterizes Italian adolescents too.

### Are There Any Differences Between Students With and Without Reading Difficulties?

Significant differences emerged between students with and without reading difficulties on total performance for Version 1, more specifically in the Evaluate subscale and for textual information. Such differences confirm our hypotheses about the disadvantage that students with reading difficulties would face when confronted with text (even if voice-to-speech technology was available to support reading). This is likely to have its origin in the differences that were found between the groups in offline reading comprehension, syntactic comprehension, verbal memory (in addition to obvious impairments in reading speed and accuracy). As to the Evaluate component, the difference emerging from the results also points to a lower level of critical appraisal and capacity to assess the reliability of online sources and choose the best source of information with respect to a specific question. This is in line with what reported by previous studies describing dyslexic students’ difficulties in understanding page layout, navigating, understand color function and complex texts overall (e.g., Kurniawan and Conroy, 2007). The differences in performance found for dyslexic students also point out that voice-to-speech support is probably not sufficient to help them overcome their comprehension difficulties. This should be taken as a suggestion to intensify research on further technological aids (see Nickerson, 2005) that could help not only decoding (which seems not to be so crucial) but especially text parsing and information collection. Use of colors is an example, but further and more sophisticated strategies could exploit the much greater flexibility of online texts and sources with respect to offline ones. Some online services, for instance, already offer the possibility to change text features in real-time according to the individual needs of dyslexic readers (e.g., changing spatial placement of text boxes and figures, types of characters used, inter-character, and inter-lines spacing etc.), and international groups are working on the development of systems able to simplify the syntactic structure of sentences.

### Are Online Reading Abilities Related to Offline Reading Skills?

The answer to this question needs some distinctions to be made between reading skills (decoding) and comprehension abilities. Offline reading comprehension is very clearly related to online reading comprehension, as emerging from both correlation and regression analyses. This confirms what already reported in the literature (Leu et al., 2011; Kiili et al., 2018). As to decoding abilities, they show very little correlations with ORCA.IT variables and they enter the regression equation for Version 2 only, together with other language skills, suggesting that some linguistic difficulty in item formulation and in the texts to be read has significantly contributed to test performance, possibly due to the presence of students with very varying levels of verbal and reading abilities (often correlated in turn). No contribution of decoding skills has been shown for the most reliable version of the test, Version 1. Indeed, it has been previously shown that the relationship between decoding and comprehension is stronger when comprehension is assessed with a cloze test, but weaker when using multiple-choice questions (Francis et al., 2005); moreover, decoding skills have more impact on comprehension for younger/less skilled readers than for older/more able ones; further, for short rather than long passages (Keenan et al., 2008). Thus, the materials used in the present study, as well as the characteristics of participants, were likely to make the test more independent of decoding.

### What Are the Predictors and Components of Online Reading Abilities (Digital Experience, Prior Knowledge, Offline Reading Comprehension, STM, WM, and Executive Skills)?

The regression analysis showed a very interesting set of underlying skills explaining a large portion (68%) of the variance for Version 1, and a different set of variables explaining a smaller proportion (36%) of variance for Version 2. Specifically, performance on Version 1 is explained by general non-verbal reasoning, prior knowledge on the topics, offline text reading comprehension, attention (especially concentration) and memory skills. This is totally consistent with what reported in published studies about the predictors and components of online reading skills (Cromley et al., 2010; Tarchi, 2010; Snowling, 2013; Kanniainen et al., 2019). A different explanation is probably necessary for the results of the regression analysis for Version 2, where linguistic difficulty seems to have been central to performance, and may also have biased the reliability and internal consistency of the results. In this case, the contribution of decoding skills, as suggested above, could be linked to general language ability (reading and language abilities being moderately associated) rather than representing an independent underlying factor. It should also be considered that the students could use text-to-speech to support reading, thus further minimizing the effect of decoding ability on text comprehension. Rather, higher-level language skills involved in text analysis and syntactic comprehension, together with attention and concentration, appear to have strongly influenced performance levels. A special mention goes to the fact that general intelligence measured by non-verbal IQ scales reveals little (and almost non-significant) contribution to online reading and comprehension skills.

It is also interesting to note that prior knowledge had a large effect in our results, differently from what was found by Kanniainen et al. (2019). Indeed, following Coiro (2011), it could be hypothesized that prior knowledge helps compensate lacking comprehension skills, but it is rather uninfluential when comprehension and online search skills are adequate; since the group of students in our study included several students with decoding difficulties, this may have strengthened the role played by familiarity with the topics.

As to the lack of effects by digital competence, we argue that general digital competence (which could be described as a more “technical” and procedural skill) as investigated in the pre-test module has no or little impact on comprehension of online information, which represents a complex, exquisitely cognitive integration and evaluation process. Indeed, such a clear-cut dissociation between procedural and processing skills may also reflect the fact that Internet searching in ORCA.IT was not real but simulated (so as to be less unpredictable, as a desired characteristic, but also less complex, an undesired side-effect) and the greater focus on information processing and comprehension than on more specific digital skills (since these are the object of existing assessment tools). The results show that the present test is in fact capable to capture such processing skills, which are, in our opinion, crucial requirements for the construction of real competence and knowledge, and relevant for integration, adaptation and success in educational and professional contexts.

## Conclusion

In conclusion, the newly developed test, especially in the most complete version (Version 1) appears to capture students’ web searching abilities and online reading comprehension in an objective, accurate and reliable way. The tool is able to highlight differences in ability between students with and without reading difficulties, not penalizing overall performance but allowing very specific weaknesses to be pointed out. Similarly, it seems to be able to capture differences due to both educational pathways (different school types) and social attitudes (differences between males and females). Even more interestingly, it shows to be clearly resting on specific cognitive and neuropsychological abilities which explain much of the total variance. Prior knowledge also influences the results, as expected. No contribution from digital competence is found, which is rather unexpected. This can be seen as a potentially positive feature of the instrument, which turns out to be rather independent of previous Internet experience and to measure more cognitively grounded processes related to information gathering, processing and communicating. Also, no influence of decoding ability emerged, possibly thanks (at least in part) to the text-to-speech facility implemented in the software.

The tool has thus the potential to be used as a screening tool to identify students who need a special training to improve their specific skills necessary for effective use of online information. It could also be useful in a clinical setting where adolescents with language and reading disorders undergo special trainings for cognitive empowerment and remediation.

## Data Availability Statement

The datasets generated for this study are available on request to the corresponding author.

## Ethics Statement

The studies involving human participants were reviewed and approved by the Scientific and Ethics Committee of the Department of Psychology of the Catholic University of Milan. Written informed consent to participate in this study was provided by the participants’ legal guardian/next of kin.

## Author Contributions

MC contributed to the conception of the study, definition of the experimental materials, performed data collection, and contributed to the writing of the manuscript. MG assisted in the definition of the experimental materials, helped in data collection, and wrote a section of the manuscript. AT performed part of the statistical analyses and participated in the interpretation and description of the results. MM assisted in the recruitment of participants. DS contributed to the definition and correction of neuropsychological tests. MV participated in the conception of the study, and organized and supervised data collection. ML took care of the conception and definition of the experimental design, assisted in the definition of experimental materials, participated in the interpretation of results, performed most statistical analyses, contributed to the writing of the manuscript, and supervised the whole study. All authors contributed to manuscript revision, read and approved the submitted version.

## Conflict of Interest

The authors declare that the research was conducted in the absence of any commercial or financial relationships that could be construed as a potential conflict of interest.

## Footnotes

1 https://responsivevoice.org/
